# Differentiation of regulatory myeloid and T-cells from adult human hematopoietic stem cells after allogeneic stimulation

**DOI:** 10.3389/fimmu.2024.1366972

**Published:** 2024-02-22

**Authors:** James M. Mathew, Jes M. Sanders, Robert Cirocco, Joshua Miller, Joseph R. Leventhal

**Affiliations:** ^1^ Comprehensive Transplant Center, Department of Surgery, Northwestern University Feinberg School of Medicine, Chicago, IL, United States; ^2^ Department of Microbiology-Immunology, Northwestern University Feinberg School of Medicine, Chicago, IL, United States; ^3^ HLA Laboratory, LeHigh Valley Health Network, Allentown, PA, United States

**Keywords:** hematopoietic stem cell transplant, transplant tolerance, myeloid derived suppressor cells, regulatory T cells, *de novo* T cell maturation

## Abstract

**Introduction:**

Donor hematopoietic stem cell (DHSC) infusions are increasingly being studied in transplant patients for tolerance induction.

**Methods:**

To analyze the fate of infused DHSCs in patients, we developed an *in vitro* culture system utilizing CD34^+^DHSCs stimulated with irradiated allogeneic cells in cytokine supplemented medium long-term.

**Results:**

Flow cytometric analyses revealed loss of the CD34 marker and an increase in CD33^+^ myeloid and CD3^+^ T-cell proportion by 10.4% and 72.7%, respectively, after 21 days in culture. T-cells primarily expressed TcR-αβ and were of both CD4^+^ and CD8^+^ subsets. Approximately 80% of CD3^+^ T cells lacked expression of the co-stimulatory receptor CD28. The CD4^+^ compartment was predominated by CD4^+^CD25^+^CD127-FOXP3^+^ Tregs (>50% CD4^+^CD127- compartment) with <1% of all leukocytes exhibiting a CD4^+^CD127^+^ phenotype. Molecular analyses for T-cell receptor excision circles showed recent and increased numbers of TcR rearrangements in generated T cells over time suggesting *de novo* differentiation from DHSCs. CD33^+^ myeloid cells mostly expressed HLA-DR, but lacked expression of co-stimulatory receptors CD80 and CD83. When studied as modulators in primary mixed lymphocyte reactions where the cells used to stimulate the DHSC were used as responders, the DHSC-lines and their purified CD8^+^, CD4^+^, CD33^+^ and linage negative subsets inhibited the responses in a dose-dependent and non-specific fashion. The CD8^+^ cell-mediated inhibition was due to direct lysis of responder cells.

**Discussion:**

Extrapolation of these results into the clinical situation would suggest that DHSC infusions into transplant recipients may generate multiple subsets of donor “chimeric” cells and promote recipient Treg development that could regulate the anti-donor immune response in the periphery. These studies have also indicated that T cell maturation can occur *in vitro* in response to allogeneic stimulation without the pre-requisite of a thymic-like environment or NOTCH signaling stimulatory cell line.

## 1 Introduction

Infusions of donor bone marrow cells (DBMC) and donor hematopoietic stem cells (DHSC) have been utilized for induction of donor specific tolerance in clinical transplantation (1–14). DHSC infusion may have a number of immunological effects (15) including the infused cells functioning as: 1) down-regulators of anti-donor immunity, 2) stimulators that lead to sensitization of the recipient, 3) responders that cause graft versus host disease (GvHD), or 4) autologous inhibitors of graft versus host responses. These potential immune effects have been studied using non-chimeric marrow from deceased donors *in vitro* (16–22), and the results suggested a sum-total strong inhibitory effect by a number of DBMC sub-populations that could overcome both responding and stimulatory effects, thereby promoting an overall state of unresponsiveness (15). However, the mechanisms by which DHSCs or DBMCs induce a tolerogenic environment have not been fully characterized.

Durable engraftment by DHSCs is required for the establishment of sustained tolerance (23–26), and such tolerance persists as long as donor cells are present within the recipient (27–29). DHSCs engraft in both the bone marrow and thymus, and DHSCs that engraft within the thymus subsequently participate in a series of tightly controlled processes that regenerate the T-cell pool after lymphodepleting, pre-transplant conditioning regimens (29). These processes consist of a series of positive and negative selection steps reliant upon thymic epithelial cells. Thus, the thymic microenvironment has been shown to be critical for T-cell maturation both *in vivo* and in *in vitro* studies utilizing thymic surrogates for *ex-vivo* T-cell generation. T-cell maturation additionally relies on specific intracellular signals, including those mediated by NOTCH receptors and their ligands, which promote *in vitro* differentiation of HSCs into lymphocytes when constitutively expressed or supplemented in cell culture (30). However, there are also processes simultaneously at play in the periphery that contribute to the tolerant state, including deletion of alloreactive lymphocytes, T cell anergy, and creation of peripherally induced regulatory cells (31–34). Studies have mostly focused on the fate of HSCs that successfully engraft, and as such little is known of the fate of DHSCs that remain in the periphery following infusion. This is of paramount importance to fully understand the mechanisms that bring about the tolerogenic effects observed from both animal and human studies, specifically in the short-term period following DHSC infusion.

Therefore, we have developed an *in vitro* culture system wherein purified adult CD34^+^ DHSCs were cultured with irradiated allogeneic cells in cytokine supplemented medium—conditions that could be similar to that following HSC infusion. We observed that DHSCs predominantly differentiate into T cell subsets and CD33^+^ myeloid cells, all of which exhibited significant immunoregulatory function. Further, the generation of new CD3^+^ T-cells indicates that neither a thymic-like environment nor a NOTCH signaling stimulatory cell line is essential for *de novo* T-cell maturation *ex-vivo*.

## 2 Materials and methods

### 2.1 Bone marrow cells

Bone marrow cells were isolated from human deceased donor vertebral bodies as described previously (19). Briefly, after thorough removal of muscular tissue and washing, the vertebral bodies were crushed and single cell suspensions were prepared. Microscopic bony fragments were removed as a pellet after a quick centrifugation to 300 x g. The bone marrow cells were then pelleted down from the supernatant by centrifugation at 300 x g for 10 min. Most of these donor bone marrow cells were used for infusion into renal and liver transplant recipients according to our experimental protocols to induce donor chimerism (15, 18, 35). However, aliquots of each were purified by Ficoll-Hypaque (Pelfreez, Brown Deer, WI) gradient centrifugation at 400 x g for 20 min. Cells were further purified into subsets as described below. Cell collections were approved under Northwestern University IRB STU00002452 and University of Miami IRB 20010146.

### 2.2 Enrichment of CD3^+^ and CD34^+^ positive cells

The CD34^+^ donor hematopoietic stem cells (DHSCs) were prepared using the CD34 isolation kit (Miltenyi Biotech Inc., Auburn, CA). Similarly, T cells were enriched using anti-CD3 coated microbeads with the MACS system via positive selection (Miltenyi Biotech Inc., Auburn, CA). Flow cytometry using appropriate monoclonal antibodies (mAbs) showed that the positively selected populations had 95 - 99% of cells expressing the target epitope. These cells were then used for *in vitro* assays.

### 2.3 Culture of DHSC to produce cell lines *in vitro*

CD34^+^ cells were stimulated with an equal number of allogeneic irradiated spleen cells or PBMC (Ax) in NAB-CM (RPMI-1640 supplemented with 15% normal AB serum, 2 mM L-glutamine, 10 mM HEPES, and 1X antibiotic-antimycotic solution; all from GibcoBRL, Gaithersburg, MD), additionally supplemented with 50U/mL rIL2 plus 50% MLR supernatant. (MLR supernatants were produced by culturing non-irradiated spleen cells or PBMC from three or more individuals in NAB-CM and harvesting the supernatant after 48 hours). The viable cells were counted using trypan blue dye exclusion at indicated intervals. Cultures were restimulated every 2 weeks with an equal number of irradiated cells from the original stimulator. The cell lines were designated as DHSC-L and were characterized phenotypically, genotypically and functionally.

### 2.4 Flow cytometry

Flow cytometric analyses was performed according to standard methods. Briefly, 0.1-0.5x10^6^ cells suspended in Dulbecco’s PBS (without Ca & Mg) supplemented with 3% normal human serum were incubated with a panel of appropriate mABS directly conjugated with one of four fluorochromes, i.e., fluorescein isothiocyanate (FITC), phycoerythrin (PE), Electro-couple Dye (ECD), phycoerythrin-cyanin 5 (PC5) and phycoerythrin-cyanin 7 (PC7) (Beckman-Coulter, Miami, FL), for 30 min at 4°C. Alternatively, Peridinin chlorophyll protein (Percp), or allophycocyanin (APC) were also used instead of PC5 and PC7. Subsequently, the cells were washed, read in a 4 or 5-color flow cytometer (Beckman-Coulter) and 1x10^4^-1x10^5^ cells were analyzed for indicated phenotypes after establishing negative population(s) with isotype controls.

### 2.5 Quantitation of T-cell receptor excision circles in DHSC-L

On indicated days the DHSC-L were further purified by sequentially depleting residual allogeneic stimulator cells and positively selecting for cells of donor phenotype using biotinylated anti-HLA class-I antibodies and streptavidin-microbeads (Miltenyi MACS system). T-cell receptor excision circles (Trecs) present in 1x10^5^ purified DHSC-L were quantified using the methods as previously described (36). In brief, real-time PCRs were performed on an ABI Prism 7700 Sequence Detection System using reagents obtained from PE-Applied Biosystems. For quantitation, each 50μl reaction contained 25μl universal master mix, 5–15ng of DNA, 200 or 450ng of each specific forward and reverse primer (36), and 0.2μM of one of the following 3 TaqMan® probes: V-Jγ^taq^, V-DJβ^taq^, or Cβ2^taq^. Each DNA sample was analyzed in triplicate at each of two concentrations for each primer/probe set. Simultaneously, real-time PCR assays were performed with dilutions of genomic DNA from purified CD3^+^ cells of normal PBMC to generate standard curves for the V-Jγ^taq^, V-DJβ^taq^ and Cβ2^taq^ amplicons. Using these standard curves, the V-Jγ, V-DJβ and Cβ copy numbers in genomic DNA isolated from 1x10^5^ purified DHSC-L were calculated. CD3^+^ cells and non-T-cells from normal PBMC were used as positive and negative controls, respectively.

### 2.6 Proliferative responses of DHSCs

Allogeneic responses of purified bone marrow or spleen cell subsets were measured using a standard ^3^[H]thymidine incorporation assay (16). Briefly, 1x10^5^ responder cells were stimulated either with 1x10^5^ irradiated spleen cells from deceased organ donors or PBMC from laboratory volunteers (having at least one HLA-DR antigen matched with the responder to mimic the institutional minimum requirement for renal transplantation) in 96-well flat-bottom plates at a total volume of 0.2 mL NAB-CM per well (see above) in triplicate at 37°C in 5% CO_2_. On the seventh day, 1 mCi ^3^[H]thymidine was added to each well. After 18 hours, the cultures were harvested using a Skatron cell harvester (Skatron, Inc., Sterling, VA), and the radioactive incorporation was monitored using a Packard-Beta counter (Packard, Meriden, CT). The data were expressed as stimulation indices (S.I) using the formula:


$$S.I.=\;\frac{CPM\;with\;experimental\;allogeneic\;stimulators}{CPM\;in\;control\;autologous\;stimulators}$$

### 2.7 Modulation of proliferation by DHSC-L

The modulatory effects of DHSC-L and purified subsets on the proliferative responses of allogeneic cells in MLR were measured using a modified ^3^[H]thymidine incorporation assay as previously published (17). DHSC-L subsets were purified with designated cell type microbeads (i.e., anti-CD4, anti-CD33, anti-CD8.) with the MACS system via positive selection (Miltenyi Biotech Inc., Auburn, CA) with average purity >95%. Lineage negative subsets (Lin-) were the remaining cells after further depletion of CD56^+^, CD14^+^, and CD19^+^ cells. 1x10^5^ responder PBMC from the individual (A) originally used as a stimulator to produce the DHSC-L were then stimulated with 1x10^5^ irradiated spleen cells from the DHSC donor in the presence of decreasing concentrations (ranging from 5 - 0.2 x10^4^) of DHSC-L subsets as modulator cells. Control cells used as modulators were either irradiated spleen cells or T cell depleted spleen cells from the same bone marrow donor. The assays were carried out in 96-well flat bottom plates and standard ^3^[H]thymidine incorporation assays were performed as above. The data were also calculated as percentage inhibition:


$$\%\;inhibition=1-\left(\frac{CPM\;with\;DHSC\;modulator}{CPM\;with\;spleen\;cell\;modulator}\right)\times \;100$$

### 2.8 Generation of new Tregs in allogeneic responders

Allogeneic or autologous CFSE-labeled responder PBMC (5x10^5^) were stimulated with PKH26-labeled and irradiated allogeneic or autologous stimulator cells (5x10^5^) in the presence of the indicated number of PKH26-labeled DHSC-L or autologous control cells as modulators. The percentages of CD4^+^CD127^-^CD25^+^FOXP3^+^ cells (total Tregs) and CD4^+^CD127^-^CD25^High^FOXP3^+^ cells (nTregs) that were newly generated in the CFSE diluted fraction of the responding cells were measured by flow cytometry after 5, 7 and 9 days. This was performed in parallel with the modulation of proliferation experiments described above.

### 2.9 ^51^Cr-release CTL assay

Chromium release assays were conducted by adding a graded number of effector cells to 5 x 10^3 51^Cr-labled target cells at different ratios of effector: target cells in triplicate in 96-well round-bottom culture plates in a volume of 0.2mL NAB-CM per well at 37°C in 5% CO_2._ Supernatants were harvested after 4 hr incubation, and the radioactive counts were measured using a Packard gamma-counter. Spontaneous release (SR) and maximum release (MR) were determined by adding target cells to wells containing 0.2mL of NAB-CM or 1% Triton X-100, respectively. Percent specific lysis was defined as:


$$\%\;specific\;lysis=\;\frac{cpm\;\left(sample\right)-CMP\;\left(SR\right)}{cpm\;\left(MR\right)-CPM\;\left(SR\right)}\;x\;100$$

### 2.10 Statistical methods

Data were analyzed using univariate and graphical methods wherever applicable. Outliers and influential observations were identified. Paired T tests were also used to compare the effect of DHSC-L vs. controls. Statistical significance was established at a two-sided alpha level of 0.05.

## 3 Results

### 3.1 Alloreactive response of DHSCs

**Figure 1:**
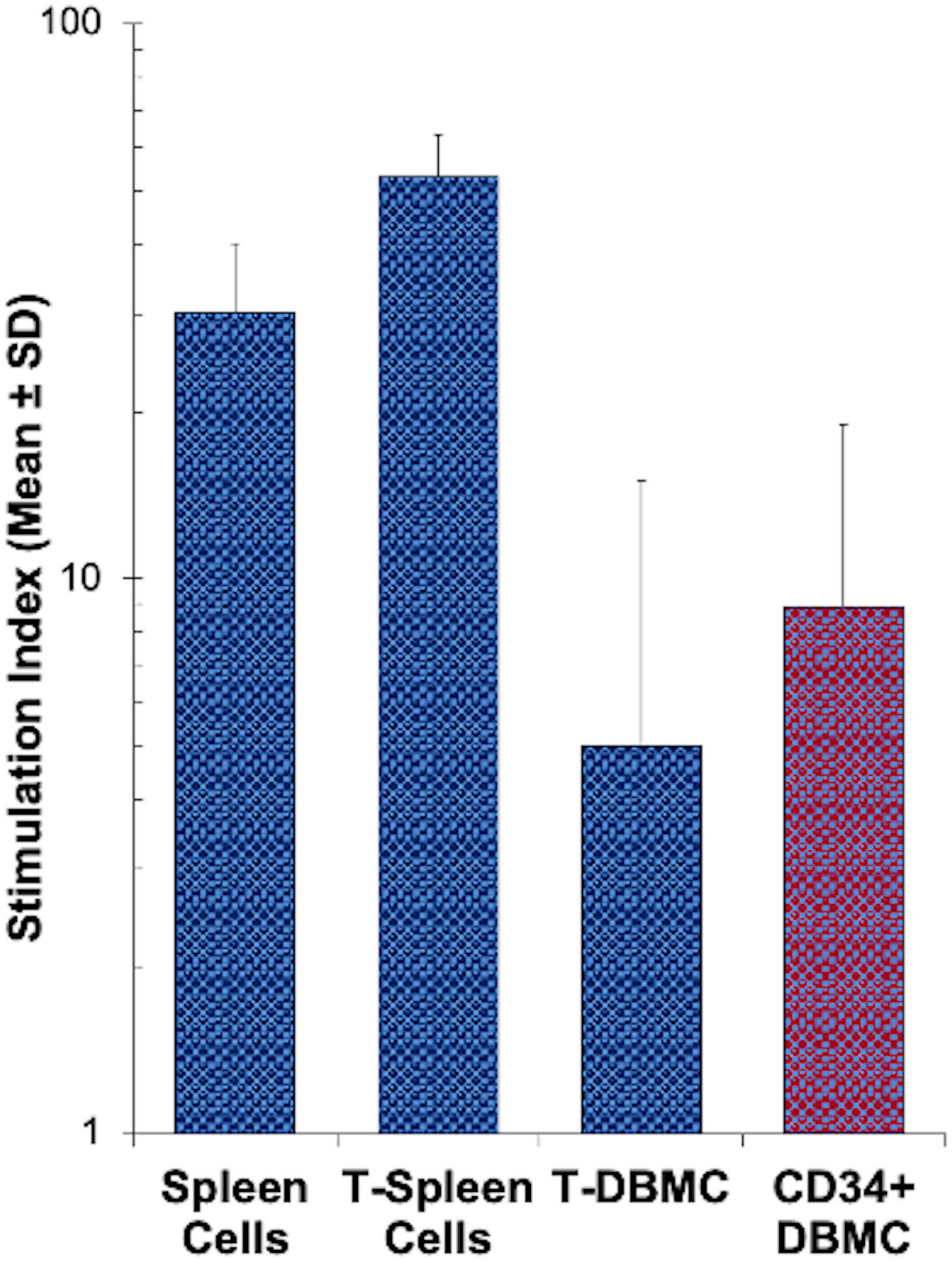
MLR response by sub-populations of human spleen or bone marrow cells: Indicated subsets of spleen cells and bone marrow cells freshly obtained from deceased donor tissues were enriched using appropriate monoclonal antibodies directly conjugated with magnetic microbeads and the MACS system. 1x10^5^ cells of each subset were stimulated with 1x10^5^ irradiated allogeneic stimulator cells and a standard ^3^[H]thymidine incorporation assay was performed on day 7. Data are expressed as stimulation index (Mean ± S.D; n=25 for CD34+ DBMC, n = 9 for all 3 other subsets), where autologous irradiated spleen cells were the controls. CD34^+^ cells from the DBMC responded to allogeneic stimulation, as strong as the bone marrow T cells.

In order to determine whether CD34^+^DHSCs are able to respond to allogeneic cells, *in vitro* alloreactive proliferative responses were measured in mixed lymphocyte reaction (MLR) cultures. As shown in **Figure 1**, CD34^+^DHSC proliferated in response to allogeneic stimulations (N=25). When expressed as a stimulation index, this proliferation was lower than the responses of spleen cells (N=9), mostly due in part to the high spontaneous proliferation of DHSC, i.e., ^3^ [H] thymidine uptakes between 2,000 - 25,000 CPM, in contrast to less than 3,000 CPM incorporated by spleen cells (data not shown). The stimulation index shown by CD34^+^DHSC was as strong as that observed with CD3^+^T-cells purified from vertebral body bone marrow from the same donor (N=9), indicating that CD34^+^DHSCs are capable of reacting to and proliferating in an allogeneic environment.

### 3.2 Differentiation of CD34^+^DHSC in culture

**Figure 2:**
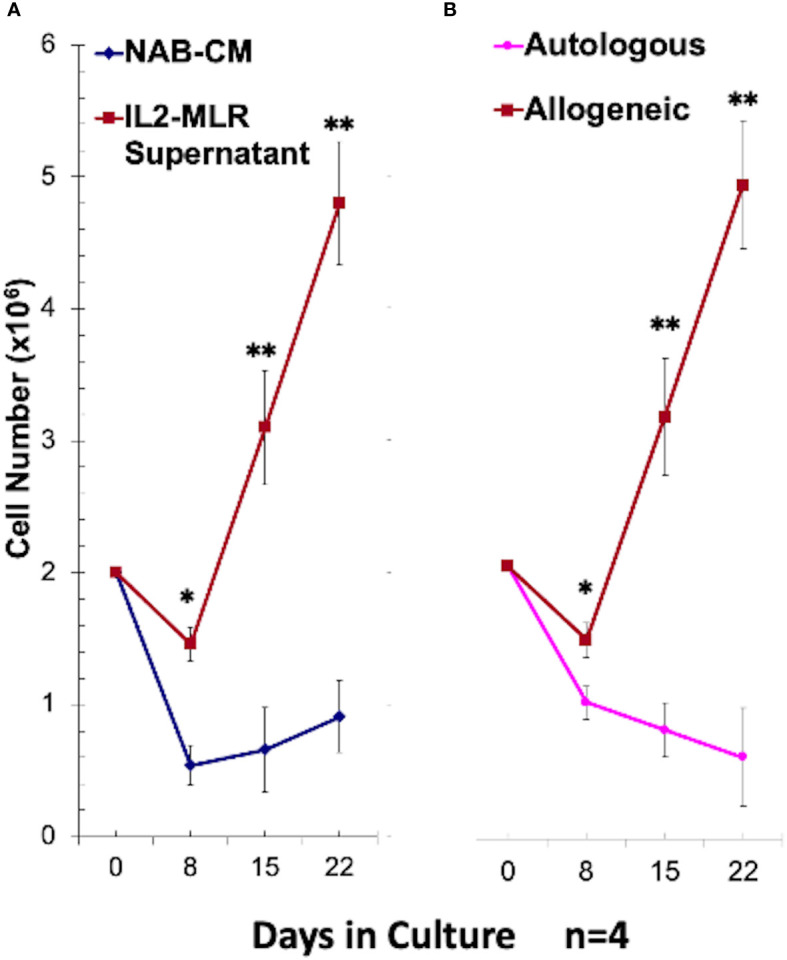
Optimal growth conditions for CD34^+^ DHSC cell line (DHSC-L) generation: CD34^+^ DHSC were cultured with an equal number of irradiated PBMC and viable cells were counted using trypan blue on indicated days (N=4). **(A)** The CD34^+^ DHSC were stimulated with irradiated allogeneic PBMC (Ax) in 15% AB serum containing RPMI-1640 medium (NAB-CM) or in NAB-CM additionally supplemented with 50U/ml rIL2 plus 50% MLR supernatant. DHSC cultures supplemented with IL2-MLR supernatant had greater cell numbers compared to NAB-CM alone at Day 8, Day 15, and Day 22. **(B)** The CD34^+^ DHSC were stimulated with irradiated autologous spleen cells or allogeneic PBMC in NAB-CM additionally supplemented with 50U/ml rIL2 plus 50% MLR supernatant. DHSC cultures stimulated with allogeneic cells had greater cell numbers compared to autologous stimulation at Day 8, Day 15, and Day 22.The resulting cell line designated as DHSC-L was phenotypically and functionally characterized in subsequent experiments. *p<0.05, **p<0.01.

To test if the above alloreactivity of CD34^+^ cells was accompanied by cellular differentiation, purified CD34^+^DHSC were cultured with irradiated allogeneic stimulator cells in cytokine rich medium with bi-weekly restimulations as described under Materials and Methods. In terms of cell proliferation, there was a 2-5 fold increase in total number of cells by day 28 (**Figure 2**). The presence of multiple cytokines in the form of 50% MLR supernatant and 50U/mL IL2 were essential for the cell expansion (**Figure 2A**) as DHSCs cultured in NAB-CM without supplementation showed minimal growth. Similarly, allogeneic stimulations were required for cell growth, and no increase in cell numbers were observed with autologous irradiated spleen cell stimulators (**Figure 2B**). These repeatedly allostimulated cultures grown in cytokine rich medium were denoted as DHSC-L.

**Figure 3:**
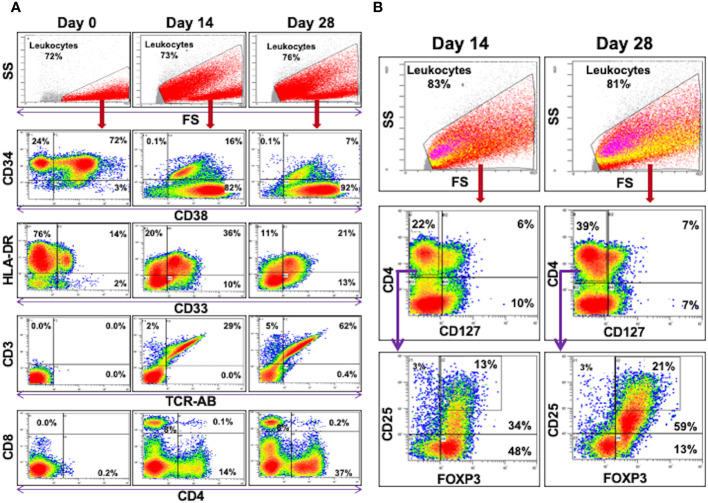
Flow Cytometric Analysis of DHSC: CD34^+^ DHSC from DBMC were cultured with allogeneic stimulators in presence of exogenous cytokines and 5 color flow analyses were performed on indicated days. The data are shown as % of cells of indicated gates. **(A)** All the data are shown as % of leukocytes. Note that the CD34^+^ cells (>96% on day 0) gradually differentiated into CD33^+^ or CD3^+^ cells, the latter into both CD4^+^ or CD8^+^ subsets**. (B)** Events in the leukocyte gate were analyzed for CD4 vs CD127 and then the CD127^-^CD4^+^ cells were further interrogated for CD25 and FOXP3 expressions. (In the top leukocyte gate, CD127^-^CD4^+^ cells are shown in yellow and the CD25^high^FOXP3^+^ cells are in pink.) CD127^-^CD4^+^CD25^High^FOXP3^+^ regulatory T cells increased proportionately over culture duration (and also in absolute numbers).

DHSC-L were characterized phenotypically using flow cytometric analysis (**Figure 3**, **Table 1**). Prior to culture, the purified cells were >95% CD34^+^ and mostly co-expressed the lymphoid marker CD38 (>70%); however, no CD3^+^ cells of either CD4^+^ or CD8^+^ subset were present (**Figure 3A**, left column, Day 0). It was observed that the CD34 marker was gradually lost from the DHSC with increasing duration of culture (95.2%, 8.4%, and 2.4%) and was accompanied by increased expression of various other cell phenotypes. At 14 days, the predominant populations of cells that were generated in the DHSC cultures were CD3^+^ T-cells (36.8%) and CD33^+^ myeloid cells (24.1%), with very little CD56^+^ NK cells (3.2%), CD19^+^ B-cells (1.6%) or CD14^+^ monocytes (2.1%) (**Table 1**). The percentage of CD33^+^ myeloid cells initially increased from 9% to 24.1% after 14 days but then gradually decreased to 19.4% by Day 28 (**Figure 3A**), even though the absolute number did not decline (data not shown). This likely resulted from the disproportionate increase in the CD3^+^ TcRαβ^+^ T-cells distributed into both CD4^+^ and CD8^+^ subsets with increasing duration of the cultures (**Figure 3A**, bottom rows). Less than 20% of CD3+ T-cells expressed either CD28 or HLA-DR, and there were no T-cells positive for CD152, CD154 and CD69 (Data not shown). Notably, proportions of CD4^+^CD127^-^CD25^+^FOXP3^+^ (Tregs) and CD4^+^CD127^-^CD25^High^FOXP3^+^ (natural Tregs) cells increased with the duration of the cultures (**Figure 3B** and bottom of **Table 1**). The majority of these Tregs were CD45RA negative and therefore presumably of an activated phenotype (data not shown). A large percentage of CD33^+^ myeloid cells expressed HLA-DR (~90%) and proportionally increased over the culture duration, but expression of co-stimulatory molecules CD80 and CD83 remained low. Interestingly, CD33^+^CD11c^+^ cell proportions gradually decreased.

**Table 1:** Subset development from allostimulated DHSC (n=7)^§^.

Cell Phenotype	Cell Subset	Day 0			Day 14				Day 28				
		Mean	±	SD	Mean	±	SD	p<	Mean		±	SD	p<
**CD34^+^**	**DHSC**	**95.2**	**±**	**2.9**	**8.4**	**±**	**10.0**	**	*2.4*		*±*	*2.9*	**
CD34^+^CD38^+^	Lymphoid DHSC	75.8	±	6.4	6.8	±	8.4	*	*2.2*		*±*	*2.9*	*
CD3^+^CD38^+^	T-cell Precursors	0.1	±	0.1	35.7	±	24.8	*	*66.3*		*±*	*11.0*	**
**CD3^+^**	**T-Cells**	**0.2**	**±**	**0.2**	**36.8**	**±**	**24.6**	*	*72.9*		*±*	*7.9*	**
CD3^+^TCR-αβ^+^	T-Cells	0.0	±	0.1	30.9	±	22.2	*	*62.2*		*±*	*5.9*	**
**CD3^+^CD4^+^**	**T-Cell Subset**	**0.1**	**±**	**0.1**	**18.1**	**±**	**11.8**	*	*26.7*		*±*	*13.5*	*
**CD3^+^CD8^+^**	**T-Cell Subset**	**0.0**	**±**	**0.0**	**13.6**	**±**	**15.1**	*	*23.4*		*±*	*20.8*	*
CD3^+^CD4^-^CD8^-^	Double Negative T-Cells	0.1	±	0.1	5.0	±	5.7	*	*7.7*		*±*	*2.3*	*
CD3^+^HLA-DR^+^	“Activated” T-Cells	0.0	±	0.0	15.9	±	3.3	**	*12.7*		*±*	*3.5*	**
CD3^+^CD28^+^	T-Cell Subset	0.2	±	0.1	11.7	±	3.0	**	*14.5*		*±*	*2.2*	**
**CD56^+^**	**NK Cells**	**0.9**	**±**	**1.2**	**3.2**	**±**	**2.5**		*4.3*		*±*	*5.6*	
CD3^+^CD56^+^	NK-T Cells	0.0	±	0.1	1.1	±	1.1		*3.0*		*±*	*5.2*	
**CD19^+^**	**B-Cells**	**1.0**	**±**	**1.6**	**1.6**	**±**	**0.9**		*0.7*		*±*	*0.5*	
**CD33^+^**	**Myeloid Cells**	**9.0**	**±**	**6.3**	**24.1**	**±**	**19.5**	*	*19.4*		*±*	*11.5*	*
CD33^+^CD80^+^	Myeloid Subset	0.1	±	0.0	3.1	±	3.0		*3.6*		*±*	*3.2*	
CD33^+^HLA-DR^+^	Myeloid Subset	8.7	±	6.1	18.6	±	17.2		*17.4*		*±*	*6.8*	*
CD33^+^CD83^+^	Myeloid Subset	0.0	±	0.0	0.2	±	0.3		*0.1*		*±*	*0.2*	
CD33^+^CD11c^+^	Myeloid Subset	8.3	±	1.3	2.9	±	2.7		*1.4*		*±*	*1.1*	
**CD14^+^**	**Monocytes**	**0.1**	**±**	**0.0**	**2.1**	**±**	**0.5**		*3.8*		*±*	*1.6*	
^Φ^ CD4**^+^**	T-Cell Subset				20.9	±	3.6	**	*36.9*		*±*	*12.3*	**
^Φ^ CD4^+^CD127^+^	T-Cell Subset				0.9	±	0.3		*0.7*		*±*	*0.4*	
^Φ^ CD25^+^FOXP3^+^ (of CD4^+^CD127^-^)	Total Tregs				51.9	±	18.4	**	*52.1*		*±*	*5.6*	**
^Φ^ **CD25^High^FOXP3^+^** **(of CD4^+^CD127^-^)**	**Natural Tregs**				**29.9**	**±**	**17.1**	**	*24.3*		*±*	*9.6*	**

^§^Data are expressed as percentage of viable leukocytes (or of CD4^+^CD127^-^ for last 2 rows). Primary cell subsets of interest are shown in bold.

^Φ^Cells were fixed and permeabilized for intracellular FOXP3 staining (n=4).

*p< 0.05 and **p<0.01 (both when compared to day 0).

### 3.3 CD3^+^ T-cells were newly generated *in vitro*

**Figure 4:**
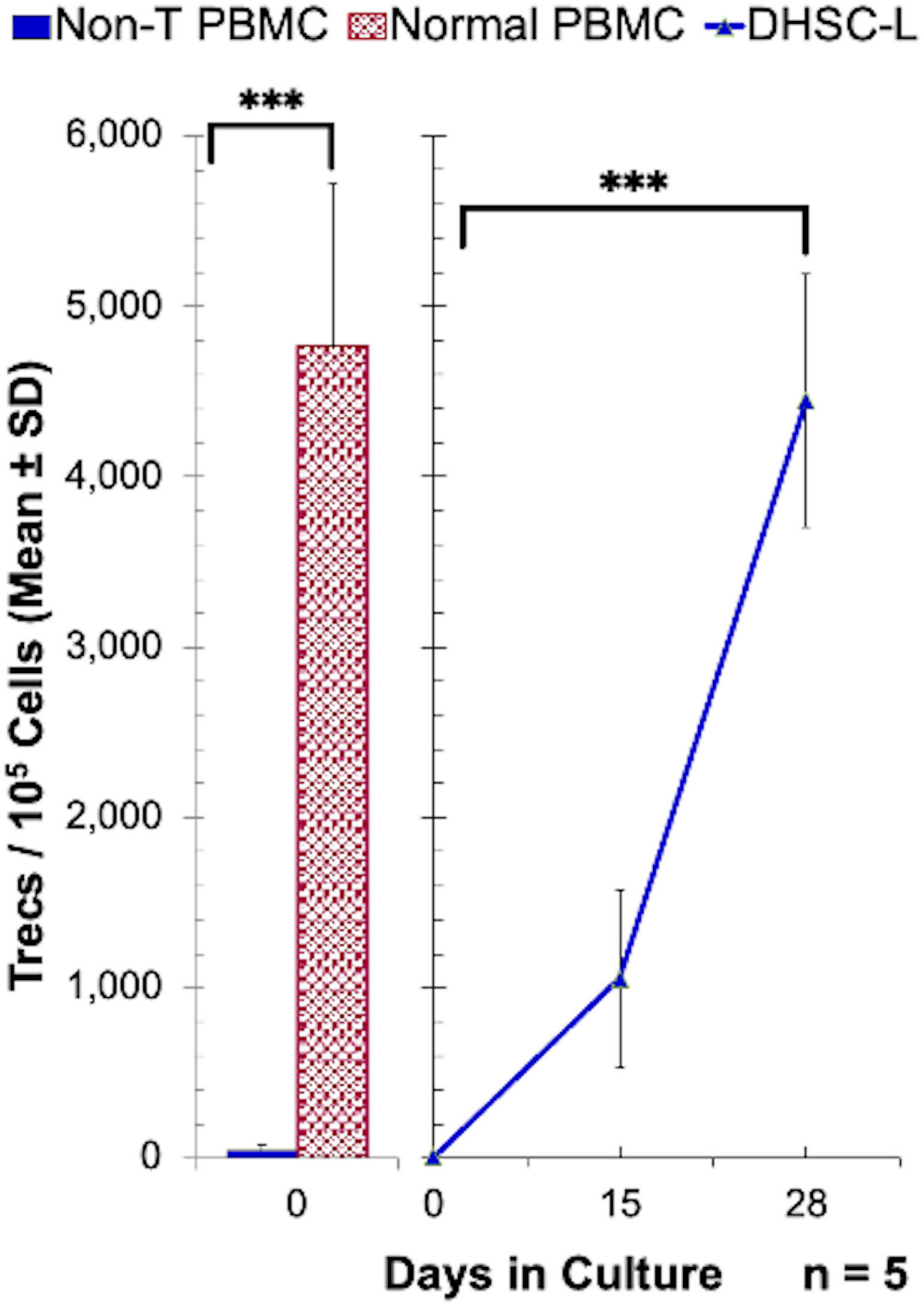
Enumeration of T-cell receptor excision circles in DHSC-L: CD34^+^ DHSC were cultured with equal number of irradiated PBMC stimulators in cytokine rich medium for indicated days. The 15 and 28 day cultures were purified by sequentially depleting any residual allogeneic stimulator cells and positively selecting for donor phenotype using biotinylated anti-HLA class-I antibodies and streptavidin-microbeads (MACS system). Then the T-cell receptor excision circles (Trecs) present in 1x10^5^ cells were quantitated using the methods as described (N=5). CD3^+^ cells and non-T-cells from normal PBMC were used as positive and negative controls, respectively. PBMCs containing T cells had greater numbers of Trecs as compared to T-cell depleted PBMCs, and there was an increase in Trecs in DHSC-L from Day 0 to Day 28. ***p<0.001.

Given the observed development of T cells, a number of experimental approaches were utilized to determine if these T cells were newly generated from CD34^+^DHSC *in vitro*. First, the possibility that a minor population of contaminating T-cells in the starting DHSC isolates expanded in culture was ruled out by: (1a) Graded proportions of purified vertebral body bone marrow CD3^+^ cells (0%, 0.1% and 1%) added on day 0 into the allostimulated CD34^+^DHSC (n=3). Subsequent monitoring on days 7 and 15 showed no enhancement of CD3^+^ cells generated in cultures with the addition of exogenous T-cells (data not shown). (1b) Conversely, any residual T-cells were eliminated from the CD34^+^DHSC by treating cells with OKT-3 and rabbit complement prior to the initiation of the cultures. Even after this drastic treatment similar proportions of T-cells as shown in **Table 1** were observed (n=3; data not shown). Second, the likelihood that T-cells observed in the DHSC-L originated from allostimulators that were resistant to irradiation was discounted by monitoring the gradual generation of CD3^+^ cells: (2a) in the CFSE diluted fractions on days 3, 7 and 14 from CD34^+^DHSC labeled with CFSE and allostimulated on day 0 (some of the data in **Table 1** were generated from these cultures), and (2b) in cultures where allostimulators used were CD3-depleted and treated with OKT-3 plus complement before irradiation (data not shown). These results indicated that the T-cells in the DHSC-L were those generated newly from the CD34^+^ DHSCs. The strongest evidence was obtained by measuring the T-cell excision circles (Trecs) in the cultures (**Figure 4**). CD34^+^DHSC were cultured with allogeneic stimulators and on days 15 and 28, cultures were further purified by sequentially depleting residual allogeneic stimulator cells and positively selecting for cells of donor phenotype. Quantitation of the Trecs revealed that there were greater levels of Trecs in DHSC-L with increasing culture duration, by day 28 nearly achieving the levels observed in normal PBMC. Importantly, cultures on day 0 showed no detectable levels of Trecs, thus demonstrating that the T-cells were newly developed from the CD34^+^DHSC cultures.

### 3.4 Immunoregulatory capabilities of DHSC-L and purified cell subsets

**Figure 5:**
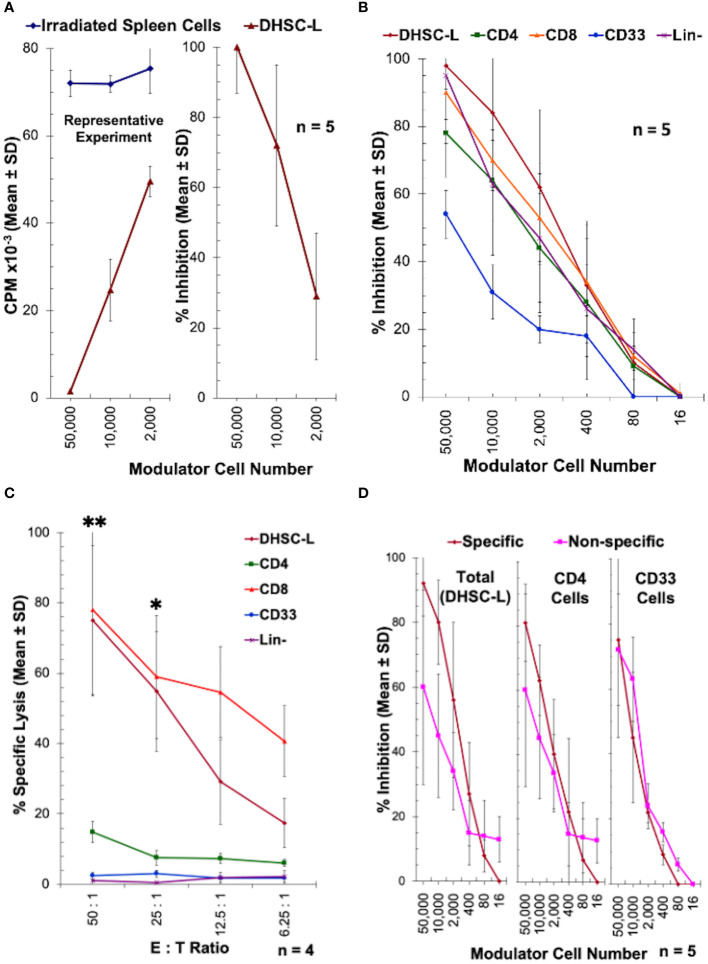
Inhibition of allogeneic MLR responses by DHSC-L: CD34^+^ DHSC were stimulated with allogeneic irradiated PBMC (Ax) in IL2-MLR supernatant medium for 28 or more days to produce DHSC-L as described in **Figure 1**. **(A)** The resulting DHSC-L were used as third component modulators in MLRs. That is, 1x10^5^ freshly prepared PBMC from the A individual **(A)** were stimulated with 1x10^5^ irradiated spleen cells (Dx) from the specific bone marrow donor in presence of indicated number of DHSC-L (or additional Dx as controls). After 7 days, a standard ^3^[H]thymidine incorporation assay was performed. Data are expressed as Mean ± SD counts per minute (CPM) from a representative experiment (left) and as % inhibition (n = 5) using the formula described in Materials and Methods. In subsequent experiments, the data are shown as % inhibition. DHSC-L inhibited the MLR responses directed against them in a dose-dependent manner. **(B–D)** *Subsets from DHSC-L:* Indicated subsets of cells were purified from the DHSC-L using appropriate monoclonal antibodies directly conjugated with magnetic microbeads (anti-CD4, anti-CD8, anti-CD33) and the MACS system. Lineage negative (Lin-) cells were those left over after other cell subset purifications and additionally depleted of any CD14^+^, CD19^+^ and CD56^+^ cells (which were only minor). **(B)** The subsets of cells were used as modulators in MLR as described in **Figure 5A** and the data are shown as percentage inhibition. DHSC-L and all subsets tested exhibited a dose-dependent inhibition in the MLR. There were no statistically significant differences between cell subsets at any of the doses tested. **(C)** The inhibition observed in **Figure 5B** could have been mediated through direct killing of the MLR responders by the modulators. To test this, purified DHSC-L subsets were used as effector cells in a 4-hour CTL assay against ^51^Chromium labeled target cells from individual A (N=4) at four ratios of effector: target cell (E:T ratio). Note that only the CD8^+^ cells and the CD8^+^ cell containing total DHSC-L and no other subsets killed the A-targets. At the two highest doses tested DHSC-L and CD8^+^ subsets had increased % lysis compared to the other subsets. **(D)** To test the specificity of MLR inhibition, PBMC from the individual A were stimulated with 1x10^5^ irradiated spleen cells (Dx) from the specific bone marrow donor or PBMC from an indifferent third party (Ix that did not have common HLA with either A or DHSC-L) in presence of indicated number of DHSC-L subsets (N=4). After 7 days, standard ^3^[H]thymidine incorporation assays were performed. There was no statistically significant difference in inhibition when allo-specific and third-party stimulators were used for DHSC-L, CD4^+^ cells, and CD33^+^ cells. *p<0.05, **p<0.01.

The rationale for infusions of DHSC into transplant patients is to induce donor specific tolerance, and as such we hypothesized that the DHSC-L developed in the *in vitro* correlate may exert immunomodulatory effects. To analyze this, DHSC-L were added as third component modulators to MLRs in which the stimulators originally used to generate DHSC-L were used as responders (A) that were then stimulated with irradiated spleen cells from the DHSC donor (Dx). **Figure 5A** shows the results as counts per minute (CPM) from a representative experiment (left) and as percentage of inhibition from five similar experiments (right). The DHSC-L inhibited the MLR in a dose-dependent manner such that even at a 1:50 modulator: responder ratio there was significant inhibition. Since the DHSC-L consisted of a mixture of cells (**Figure 3**, **Table 1**), the predominant subsets were purified, and their individual inhibitory capacity was tested in similar MLRs at broader modulator:responder ratios (**Figure 5B**). The lineage negative (Lin-) cells consisted of the remaining cells after depleting CD3^+^, CD56^+^, CD14^+^, CD33^+^, and CD19^+^ cells. As before, the DHSC-L inhibited MLRs in a dose dependent manner, and the purified cell subsets showed similar inhibitory capabilities (**Figure 5B**).

The inhibition mediated by the DHSC-L subsets could be through true inhibition or via direct killing of the responder cells allogeneic to the DHSC-L. In order to distinguish between these two possibilities, the purified DHSC-L subsets were used as effector cells against PHA-blast target cells from the A-responders (used as stimulators in generating the DHSC-L, and as the responders in the inhibition assay) in a conventional 4-hour ^51^Chromium release assay (**Figure 5C**). Only the CD8^+^ subpopulation as well as the total DHSC-L that contained the CD8^+^ cells caused the lysis of A-target cells. This indicated that the inhibition mediated by the CD8^+^ subset (**Figure 5B**) was due to the killing off of the allogeneic MLR responder cells.

In order to test the specificity of the inhibition mediated by DHSC-L, DHSC-L and their CD4^+^ and CD33^+^ subsets were added as third component modulators to MLRs with A-responders stimulated either with irradiated spleen cells from the DHSC donor or from a third-party donor having HLA completely mismatched with both the responder and the donor. As shown in **Figure 5D**, the DHSC-L subsets inhibited the responses against both the donor and third party to a similar extent. Further, the MLR responses of donor spleen cells (autologous to the DHSC-L) as well as the third party stimulated with irradiated A cells (used in generating DHSC-L) were similarly inhibited by the DHSC-L subsets (data not shown). Taken together, these results indicated that the inhibition mediated by the DHSC-L was donor non-specific.

## 4 Discussion

There is substantial evidence that infusions of donor bone marrow cells (DBMC) and donor hematopoietic stem cells (DHSC) can induce tolerance in clinical transplantation (1–14, 37). Acceptance of an allograft is possible due to the establishment of chimerism, and bone marrow infusion is known to lead to varying degrees of chimerism depending on the recipient pre-conditioning regimen (38). Full chimerism increases the risk of developing GvHD given the requirement for total replacement of recipient hematopoietic elements with donor HSCs, and as such more attention has been placed towards pre-operative conditioning regimens that result in mixed chimerism, i.e., both recipient and donor cells coexist simultaneously with mutual non-responsiveness. Studies attempting to better characterize the mechanisms by which DHSCs result in allograft tolerance have shown that sustained, mixed chimerism relies on thymic engraftment by DHSCs following transplantation (23), but there still exists a need to better understand the role of the periphery in maintaining a tolerogenic environment as even transient mixed chimerism can allow for long term acceptance of a renal allograft in non-human primates (39–41). More specifically, the terminal phenotype and functional capabilities of infused DHSCs that may remain in the periphery or undergo differentiation prior to thymic engraftment should be investigated to elucidate their contribution to such tolerant states.

Our previous observation (19) that CD34^+^ cells responded to allogeneic stimulation and proliferated in mixed lymphocyte reactions (**Figure 1**) (19) prompted us to further investigate the characteristics of cultured DHSCs through the use of our *in vitro* culture system. Purified adult CD34^+^ DHSCs were cultured with irradiated allogeneic cells in cytokine supplemented medium, a condition that could be similar to that following HSC infusion *in vivo*. It was observed that CD34^+^ cells not only expanded in culture (**Figure 2**) but also differentiated into multiple subsets. The predominant cell type was CD3^+^ T cells, of both CD4^+^ and CD8^+^ subsets, with low expression of CD28 and HLA-DR. Within the CD4^+^CD127^-^ subset, >50% were CD25^+^FOXP3^+^ with 25—30% of CD4^+^CD127^-^ cells exhibiting the CD25^high^FOXP3^+^ phenotype (**Figure 3**, **Table 1**). Importantly,<1% of all leukocytes were CD4^+^CD127^+^. The second most prevalent cell type was CD33^+^ myeloid cells, the majority of which expressed HLA-DR with little to no expression of co-stimulatory molecules CD80 and CD83. Functionally, the newly, differentiated cells were strongly immunoregulatory but lacked donor specificity (**Figure 5**).

The primary cell population that developed *in vitro* was CD3^+^ T-cells (**Figures 3**, **4**, **Table 1**). In humans, lymphoid progenitors develop in the bone marrow, but the maturation of these progenitors into T cells requires a series of highly coordinated processes that take place in the thymus (37, 42). Common lymphoid progenitors migrate to the thymus, transition through a series of CD4^/^CD8 double negative stages, and eventually become committed to either a terminal CD4^+^ or CD8^+^ phenotype following interaction with NOTCH receptor ligands and thymic epithelial cells (TECs) (30). In fact, TECs express high levels of NOTCH ligands, specifically Delta-like 4 (Dll4) (43). Thus, it is accepted that these two factors are essential for *de novo* T cell generation and repopulation following HSC transplantation. As an individual ages, the thymus involutes and secondary lymphoid organs take on a larger role in the maturation of T cells whereby lymph node stromal cells and dendritic cells function analogously to TECs (44). In an effort to develop new methods of *ex-vivo* T-cell lymphopoiesis, numerous studies have investigated the use of thymic surrogates, such as 3-D microenvironments, or artificial presentation of developmental signaling molecules (i.e., NOTCH-ligands) [Reviewed in (30)]. These investigations have shown that T cell lymphopoiesis can occur in the presence of specific 3-D microenvironments acting as a thymic surrogate (30), and also with ectopically expressed NOTCH-ligands (45–47). In our study, we observed that the differentiation of HSCs into functional CD4^+^ and CD8^+^ T cells can occur *in vitro* in the absence of both a semi-solid microenvironment and such stimulatory cell-lines (i.e., ectopically expressed or bead-bound Dll4). The only requirement was cytokine supplementation (in the form of MLR supernatant and IL-2) and allogeneic stimulation.

Multiple clinical studies have illustrated the donor specific functions of regulatory cells following combined solid organ and HSC transplantation (13, 48, 49) forming the basis for why HSC transplantation is of interest in tolerance protocols. However, in the present study we did not observe donor-specific immunoregulatory properties. Instead, the modulatory effects of DHSC-L, including the CD4^+^ and CD33^+^ subsets, were non-specific as DHSC-L downregulated proliferative responses to indifferent third-party stimulators to a similar extent as to autologous donor-specific stimulators (A + Dx or Ix + DHSC-L). We also observed the DHSC-L strongly inhibited MLRs in which autologous splenic responder cells were stimulated with irradiated allogeneic PBMCs that were used to generate the DHSC-L or indifferent third-party (D + Ax or Ix + DHSC-L) (data not shown). As we have shown previously, DHSC-L exhibited features of both myeloid derived suppressor cells (MDSCs) and veto cells, but earlier studies did not further explore the lineage markers or cytotoxic function of cultured DHSC-L (19). Here, we provide additional data that may further support those findings. Veto cells are immunoregulatory cells that are important in maintaining tolerance in the setting of HSC transplantation (22). Upon recognition of an MHC:peptide complex by a recipient responder cell, the responder cell is destroyed by the veto cell. CD8^+^ T cells are probably the most potent of veto cells, as they can destroy both recipient anti-donor cells and also donor anti-recipient cells (32, 50, 51). In the present study, we demonstrate the specific lysis of responding allogeneic (A) cells by the CD8^+^ subset present in the DHSC-L (**Figure 5**), and we have previously shown that the immunoregulatory function of these cells was contact dependent (19). Although we did not specifically test for donor specificity of the CD8^+^ subset, our data taken together suggests the CD8^+^ subset in our system may exhibit veto-cell like functions.

Within the CD4^+^ compartment, cells were predominated by CD4^+^CD127^-^CD25^+^FOXP3^+^ Tregs (~52% of the CD4^+^ population), with<2% of CD4^+^ T cells expressing CD127. Although CD127 expression is downregulated following T cell activation, levels can subsequently increase in effector and memory T cells but not in FOXP3^+^ Tregs (52). Furthermore, CD4^+^CD127^-^ cells, regardless of FOXP3 expression, have been shown to be suppressive by nature (53). Liu et al. demonstrated that traditional Tregs, CD4^+^CD127^-^, CD4^+^CD127^-^CD25^-^, and CD4^+^CD127^-^CD25^+^ cell subsets all suppressed the alloimmune response and were anergic (53). We did not perform similar cell sorted analyses; however, the CD4^+^ suppression observed in our study was likely a combination of traditional Tregs and the CD4+CD127- cell populations. Surprisingly, this inhibition was found to be non-specific. Multiple groups, including our own, have generated antigen specific Tregs through antigen education with stimulation of Tregs with antigen presenting cells (APCs). Thus, we thought this would be the case in our DHSC-L cultures. We speculate the lack of donor specificity may be due to either 1) Tregs were generated that mostly recognized MHC:peptide complexes from the “recipient” (irradiated and cryopreserved allogeneic cells used for stimulation) as there was low proportions of donor-derived APC populations (**Table 1**) or 2) the timing and frequency with which we stimulated DHSC did not allow for adequate antigen education.

The vast majority of CD33^+^ cells expressed HLA-DR with little to no expression of CD80 and CD83. It is unclear exactly what this cell population represents, but we hypothesize these cells may be an intermediate stage APC or early dendritic cell. After exposure to foreign antigens, dendritic cells (DCs) process and present these antigens via MHC-I and/or MHC-II, migrate to secondary lymphoid organs and initiate a T cell response. During this time, DCs transition from an immature to mature state, coinciding with increased expression of co-stimulatory molecules CD80, CD86, and CD83 (54). DCs can also exist in a “semi-mature” state, whereby they have been exposed to antigen but are either functionally or phenotypically incomplete. This has been described in the tumor microenvironment and is thought to contribute to intratumor T cell anergy and suppressed anti-tumor responses (54–56). These intermediate DCs may also promote the expansion of regulatory cells as it has been shown both in cancer and transplant immunology that immature DC subsets are correlated with increased levels of Tregs. However, we cannot definitively say that this population of CD33^+^ cells are DCs, and in fact, we did not observe a proportional increase in CD11c^+^ myeloid cells within the entire CD33^+^ population (**Table 1**). This could be a result of 1) skewed differentiation towards CD3^+^ T cells, 2) allogeneic stimulation resulted in upregulation of HLA-DR prior to terminal DC differentiation and thus, these cells represent an early DC precursor or 3) repeated allogeneic stimulation of DHSCs results in downregulation of CD11c. Future mechanistic studies should elucidate the exact identity and function of this CD33^+^HLA-DR^+^ myeloid subset as it may have important implications for downregulating both host v donor and donor v host alloimmune responses.

A small percentage of DHSC-L also exhibited some features of MDSCs, which have been shown to be non-specific immunoregulatory cells both *in vitro and in vivo* (57–59). Classically, MDSCs are phenotypically classified as CD33^+^HLA-DR^-^CD11b^+^. Although we did not specifically test for CD11b, a small proportion of CD33^+^ cells were HLA-DR^-^ and increased over culture duration (**Figure 3**). Data regarding specific vs non-specific immunoregulation by MDSCs in both humans and animal models is incomplete, and much of our understanding about their function stems from studies in cancer models. In transplantation, levels of MDSCs have been found to be increased in the peripheral blood of patients following kidney transplantation (60–62). Additionally, pro-inflammatory states can drive the differentiation of HSCs into MDSCs (63–65), such as that utilized in our culture system and also those observed following solid organ and/or HSC transplantation. More importantly, MDSCs can induce the formation of Tregs, with increasing levels of MDSCs being correlated with greater numbers of Tregs in renal transplant recipients (62, 66). It has been additionally suggested that the immunosuppressive functions of MDSCs are driven primarily through the induction of new Tregs (67, 68). This, combined with increased proportions of the CD33^+^HLA-DR^+^ subset described above, may account for the steadily increased proportion of Tregs observed in our culture system and also the development of newly generated Tregs in the CSFE-labeled, responder PBMCs used in MLRs.

Finally, senescence of HSCs occurs in response to various stimuli including aging, chronic inflammation, or oxidative stress (69, 70). During this process, HSCs undergo a series of changes including decreased regenerative capacity, myeloid-skewed differentiation, reduction in clonal diversity, and functional alteration. Consequences of this have primarily been studied in small animal models, but it is thought these functional changes and myeloid-based differentiation lead to ineffective immune responses in older patients (71). In contrast, our cultured DHSCs possessed regeneration capacity, evidenced by the marked expansion over 21 days in culture, demonstrated lymphoid-skewed differentiation, and retained functional properties (i.e., regulation of the alloimmune response in MLRs).

Extrapolation of the results to the clinical situation suggests that a proportion of DHSCs upon infusion may undergo a series of differentiations in the periphery in response to 1) allogeneic stimulation and 2) pro-inflammatory cytokines, allowing them to mature into immunoregulatory cells. As such, it is likely both short term, non-specific immunoregulation and long-term, donor specific tolerance are necessary for the achievement of sustainable, donor specific hypo- or unresponsiveness following concomitant HSC and solid organ transplantation. This study also demonstrates that functional T cells can be generated in the absence of a thymic-like environment and NOTCH-signaling stimulatory cell lines *in vitro*, providing a potential new method of *ex-vivo* DHSC expansion for tolerance induction. In depth characterization of the cytokines and growth factors responsible for this differentiation will need to be conducted as we did not analyze the inflammatory milieu in DHSC cultures. For example, the disproportionate increase in T cell populations observed in our study may have been influenced by the addition of IL-2. A more balanced or even a myeloid skewed differentiation could be observed if IL-2 is removed or replaced with other cytokines. These studies will be crucial for complete understanding of the mechanisms by which DHSCs may promote peripheral tolerance in the early post-transplant period and could provide evidence as to why some subjects successfully engraft and achieve durable tolerance.

## Data availability statement

The raw data supporting the conclusions of this article will be made available by the authors, without undue reservation.

## Ethics statement

The studies involving humans were approved by Northwestern University IRB (STU00002452); University of Miami IRB (20010146). The studies were conducted in accordance with the local legislation and institutional requirements. The participants provided their written informed consent to participate in this study.

## Author contributions

JMM: Conceptualization, Data curation, Investigation, Methodology, Writing – original draft, Writing – review & editing, Formal analysis. JS: Writing – original draft, Writing – review & editing. RC: Data curation, Writing – original draft, Writing – review & editing. JM: Conceptualization, Formal analysis, Writing – original draft, Writing – review & editing. JL: Data curation, Writing – original draft, Writing – review & editing.
